# An Edge Transfer Learning Approach for Calibrating Soil Electrical Conductivity Sensors

**DOI:** 10.3390/s23218710

**Published:** 2023-10-25

**Authors:** Yun-Wei Lin, Yi-Bing Lin, Ted C.-Y. Chang, Bo-Xun Lu

**Affiliations:** 1 College of Artificial Intelligence, National Yang Ming Chiao Tung University, Tainan 711, Taiwan; boxunlu.ai09@nycu.edu.tw; 2 College of Humanities and Sciences, China Medical University, Taichung 406, Taiwan; 3 Miin Wu School of Computing, National Cheng Kung University, Tainan 701, Taiwan; 4 College of Computer Science, National Yang Ming Chiao Tung University, Hsinchu 300, Taiwan; 5 Department of Computer Science and Information Engineering, Asia University, Taichung 413, Taiwan; 6 Research Center for Information Technology Innovation, Academia Sinica, Taipei 115, Taiwan; 7 Quanta Computer Co., Ltd., Taoyuan 333, Taiwan; ted.chang@quantatw.com

**Keywords:** artificial intelligence, electrical conductivity, farming sensors, Internet of Things (IoT), Random Forest, sensor calibration, XGBOOST

## Abstract

Smart agriculture utilizes Internet of Things (IoT) technologies to enable low-cost electrical conductivity (EC) sensors to support farming intelligence. Due to aging and changes in weather and soil conditions, EC sensors are prone to long-term drift over years of operation. Therefore, regular recalibration is necessary to ensure data accuracy. In most existing solutions, an EC sensor is calibrated by using the standard sensor to build the calibration table. This paper proposes SensorTalk3, an ensemble approach of machine learning models including XGBOOST and Random Forest, which can be executed at an edge device (e.g., Raspberry Pi) without GPU acceleration. Our study indicates that the soil information (both temperature and moisture sensor data) plays an important role in SensorTalk3, which significantly outperforms the existing calibration approaches. The MAPE of SensorTalk3 can be as low as 1.738%, compared to the 7.792% error of the original sensor. Our study indicates that when the errors of uncalibrated moisture and temperature sensors are not larger than 8.3%, SensorTalk3 can accurately calibrate EC. SensorTalk3 can perform model training during data collection at the edge node. When all training data are collected, AI training is also finished at the edge node. Such an AI training approach has not been found in existing edge AI approaches. We also proposed the dual-sensor detection solution to determine when to conduct recalibration. The overhead of this solution is less than twice the optimal detection scenario (which cannot be achieved practically). If the two non-standard sensors are homogeneous and stable, then the optimal detection scenario can be approached. Conventional methods require training calibration AI models in the cloud. However, SensorTalk3 introduces a significant advancement by enabling on-site transfer learning in the edge node. Given the abundance of farming sensors deployed in the fields, performing local transfer learning using low-cost edge nodes proves to be a more cost-effective solution for farmers.

## 1. Introduction

**Figure 1:**
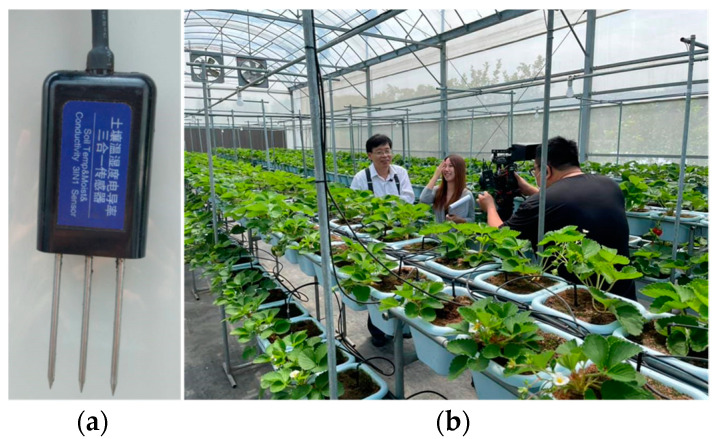
(**a**) A 3-in-one sensor and (**b**) the Bao strawberry greenhouse.

The ability of soil to conduct electrical current indicates drainage, the amount of nitrogen usage, rooting depth, water holding and cation-exchange capacity in a farm field. Such ability is typically measured through electrical conductivity (EC) [1,2]. In smart agriculture [3], we utilize Internet of Things (IoT) technologies to enable low-cost EC sensors to support farming intelligence [4,5]. Figure 1a illustrates a three-in-one sensor for the temperature, the humidity and the EC. Several three-in-one sensors are deployed in a strawberry greenhouse, as shown in Figure 1b. In this greenhouse, the fertilizer drippers are controlled through real-time AI prediction with inputs from the farming sensors.

Because of aging and changing weather and soil conditions, EC sensors are prone to long-term drift over years of operation. Therefore, they must be recalibrated on a regular basis to guarantee data accuracy.

Most smart farming studies do not consider in-field EC sensor calibration; instead, they focus on offline (laboratory) calibration, which is expensive and interrupts smart farming operations. In our previous studies [6,7,8], we addressed this issue by employing a table lookup method.

Specifically, we have developed a series of techniques for in-field EC sensor calibration called SensorTalk generations 1–3. The generations of SensorTalk were transitioned in chronological order. In the first SensorTalk generation [6,7], we designed a failure detection and calibration framework. The SensorTalk framework automatically detects the failures of many farming sensors and actuators through mutual tests among them. SensorTalk also uses a lookup table mechanism to calibrate the aging sensors. Following the cloud computing paradigm, computing tasks are offloaded from the IoT devices to the SensorTalk server in the cloud to reduce energy consumption and improve the performance of the farming IoT devices. To be more specific, the first two generations of SensorTalk use a cloud-based dashboard to detect sensor/actuator failures and calibrate the aged sensors semi-automatically.

The second generation (SensorTalk2) [8] is an extension of SensorTalk in redundancy. In this approach, nine EC sensors installed in the Murata redundant-sensor package [9] are used in a voting procedure to generate the correct EC values. When a sensor in the package is aged, calibration is automatically conducted locally to fix the aged sensor through the use of other correct sensors through the same lookup table approach of SensorTalk. SensorTalk2 is an edge computing solution that performs calibration through local redundant sensors in the Murata package [10,11,12,13].

After optimization, the accuracy of the lookup table is limited to 96%. To address this main weakness and research gap, we propose SensorTalk3 to replace the lookup table mechanism in SensorTalk with AI mechanisms. In-field experiments indicate that SensorTalk3 significantly improves the accuracy of SensorTalk, achieving rates higher than 98%. Previous approaches need to train the calibration AI models in the cloud. A major contribution of SensorTalk3 is the ability to perform transfer learning in the edge node. Since there are many farming sensors in the farm field, it is more cost-effective to perform transfer learning locally with inexpensive edge nodes for the farmers. Moreover, the process of calibrating a sensor and achieving accurate values is further delayed when employing transfer learning in the cloud. This delay results in the sensor producing inaccurate readings during the calibration period. Thankfully, by leveraging SensortTalk3’s transfer learning capabilities in the edge node, we can effectively address this problem.

The paper is organized as follows. Section 2 surveys the related work; Section 3 elaborates on the SensorTalk3 approach; Section 4 describes the AI models; Section 5 evaluates the prediction performance of SensorTalk3.

## 2. Related Studies

This section surveys the previous studies on EC sensors and sensor calibration.

### 2.1. EC Sensors

This subsection overviews the related work for EC sensor designs. An EC sensor is typically implemented with four electrodes constructed from conductive materials such as graphite, stainless steel, or platinum (Pt). In this design, two outer electrodes are built apart to pass a current produced by conductive ions (such as metals and salts in soil). The circuit uses a sinusoidal AC voltage source generated based on the theory of the Wien bridge oscillator. A low oscillation frequency (1 Hz) is selected so that the impedance depends mainly on resistance and less on capacitance or inductance. The circuit eliminates any DC offset to avoid electrode polarization. Two inner electrodes are constructed to measure the resulting current. The conductive ions between the outer electrodes create a current path, where the measured conductivity implies the ionic concentration of the soil. In this paper, conductivity is measured in Deci Siemens (dS). When using a contacting conductivity sensor, conductivity cell geometry affects the conductivity reading. In order to ensure the standardization of EC measurements, units of specific conductivity are used. Equation (1) gives the equation for specific conductivity $\sigma_{b}\;$measured in dS per meter (dS/m):

(1) $$\sigma_{b}=\sigma_{m}\kappa =\frac{\sigma_{m}L}{A}\;$$

where A is the surface area of an outer electrode and L is the distance between the outer electrodes. The cell constant κ (i.e., the volume between the two electrodes) is computed as L/A. The measured conductivity $\sigma_{m}$ (the measured current divided by the applied voltage) is multiplied by the cell constant to determine the specific bulk conductivity $\sigma_{b}$ of the soil, i.e., the EC of the undisturbed matrix of soil, water and air. 

An example of EC sensor package is the Murata Model LT5006. The Murata redundant sensor package also includes the sensors for moisture (Volumetric Water Content or VWC) and temperature, which can be used in both the soil and water at same time.

The $\sigma_{b}$ range of the Murata EC sensor is 0–5 dS/m and the resolution is 0.001 dS/m. To protect the electrodes of the EC sensor from corrosion, it is essential to use low voltage and highly corrosive-resistant materials.

It is also important to know the soil pore water EC ($\sigma_{w}$; the EC of the water in the pore spaces of the soil) as an indicator of the solute concentration. The $\sigma_{w}$ value is calculated using some parameters measured using the Murata moisture sensor. To achieve high accuracy, sensor calibration is compensated by considering the temperature dependence. The relationship between $\sigma_{w}$ and $\sigma_{b}$ is given in Equations (2) and (3).

(2) $$\sigma_{w}=\frac{\epsilon_{w}\sigma_{b}}{\epsilon_{b}-\epsilon_{b}^{*}}$$

where $\epsilon_{b}$ is the bulk soil dielectric permittivity that can be measured through the application of Equation (1). In Equation (2), $\epsilon_{b}^{*}$ is an offset, which is the real portion of the dielectric permittivity when $\sigma_{b\;}$= 0. In [14], $\epsilon_{b}^{*}$ = 4.1 is recommended as a generic offset. In Equation (2), $\epsilon_{w}$ is expressed as

(3) $$\epsilon_{w}=80.3-0.37\left(T_{s}-20\right)$$

where $T_{s}$ is the soil temperature (°C) measured by the soil sensor co-located with the bulk EC measurement in the Murata package.

There are several state-of-the-art IC designs for EC sensors. Based on Equation (2), the study in [15] fabricated a redundant sensor package for measuring pH, EC and temperature using compatible CMOS technology on a Si chip where the chip size is 5 mm × 5 mm. An EC sensing area using Pt electrodes is deposited on the Ti/Al electrode. The fabricated redundant sensor package showed good performance in a 6-day experiment. The study in [16] presents a self-sustained soil EC sensing IC using the concept of Equation (2). With a wide-range DC–DC converter, the system simultaneously measures the conductivity of the soil and harvests energy. This IC was fabricated with the 0.18µm CMOS technology. Using the carbon and zinc electrodes, the input voltage of the soil cell ranges from 0.8 to 1 V. The soil EC is measured from 0.114 to 0.744 dS/m in a resolution of 0.00568 dS/m per bit.

### 2.2. Sensor Calibration

Based on the lookup table, SensorTalk and SensorTalk2 use standard sensors to produce a calibration table for an aged sensor. Details of lookup table calibration can be found in [6,7,8] and the references therein.

In [17], the authors surveyed the machine learning techniques for the calibration of air quality monitoring sensors. They identify several open research challenges. Specifically, low-cost air quality sensors suffer from cross-sensitivities between different ambient pollutants, and their accuracy degrades over time. They are also affected by external factors, including weather changes, traffic, and human behavior. This survey indicated that periodic in-field recalibration using machine learning is promising to recover the accuracy of the sensors. SensorTalk3′s experience on EC sensor calibration has shown consistent conclusions.

In [4], in situ blind calibration for sensor network monitoring was proposed, requiring neither physical intervention nor identical ground-truth signals. A multioutput Gaussian process (MOGP) was used to model the spatial-temporal distribution of the measure and drift to remove irrelevant short-term fluctuations. MOGP also decomposes the drift from long-term trends. The algorithm was evaluated on a real-world dataset of strain sensors. This study did not take advantage of the knowledge of other types of sensors as SensorTalk3 does.

Based on extreme learning machine and projection onto convex sets, a method [18] was proposed to calibrate electronic nose drift under long-term working conditions for both recognition and regression applications specific to isopropanol and acetone gases. In [19], the authors proposed automatically calibrating a large number of barometer sensors, which uses a low-power barometer on a smartphone without requiring reference points or any manual operation. However, this work requires user encounter detection to conduct peer-to-peer calibration, which calibrates all barometers by solving a minimum dominating set problem. In a field experiment, this approach yields an accuracy of within 0.1 hpa in 82% of cases. User encounter detection cannot be guaranteed in general scenarios.

The accuracy of a gas sensor may be affected by other gases. Such interference is called cross-sensitivity. Cross-sensitivity can cause an unwanted effect on the sensor, which may be a positive response, negative response or inhibition. In [20], the authors designed a fast informed (Semi-) non-negative matrix factorization method to solve in situ calibration of cross-sensitive sensors. In these gas sensors, the readings of a sensor depend on the readings of other sensors. The approach was studied under simulation. It is not clear if the approach works in real environments.

In [21], the authors investigated metal oxide (MOx) sensors for detecting indoor Benzene, Toluene, Ethylbenzene, and Xylene gases. Since temperature and humidity can easily affect the MOx sensors, calibration is conducted through machine learning, including artificial neural network, non-linear curve fitting and linear regression. This approach was validated using three operating points of temperature/humidity in the laboratory. The experiments indicated that the proposed approach reduces 73% of the temperature and humidity impacts on the reading variation of the MOx sensors. Unlike the study in [21], SensorTalk3 does not eliminate the effects of temperature and humidity. Instead, we utilize the humidity (moisture) and temperature to improve the accuracy of soil EC sensors in farming conditions without manually setting the operation points of temperature/humidity in the laboratory.

By using a metal oxide semiconductor gas sensor as an example, the study in [22] showed how to calibrate the readings in temperature cycled operation, which measure mixtures of artificial room air containing several volatile organic compounds and quantifying formaldehyde. The study attempted to minimize the calibration time through preprocessing of the training data. Through steady-state detection, the labeled valid data points are added to the dataset as compared to a time-consuming manual annotation. By reducing 50% of the original data, the preprocessed data can still train ResNet neural networks to produce errors 25% smaller than the errors defined by the WHO.

The study in [23] utilized deep learning to investigate the effects of weather in both drifting and sensor measurements. A procedure was designed to generate simulated emission and dispersion of PM 2.5 and PM 10. The study in [24] proposed a sensor calibration method for PM2.5, which uses a domain adaptation technique to reduce the calibration time. Temperature, PM10, and humidity are used as the features of the deep learning models for predicting the PM2.5 values. The results show that both proposed models in [23,24] reduce the calibration error. However, they were not validated against the real data.

Most of the above studies attempt to calibrate the gas sensors by eliminating the cross-sensitivity effects caused by humidity and temperature. Unlike these studies, SensorTalk3 takes advantage of humidity and temperature to improve the calibration accuracy of the EC sensors.

The study in [25] developed a multivariable model, employing radial basis function artificial neural network to estimate soil EC based on various factors. Laboratory tests yielded a high R2 of 0.99 and RMSE of 0.005 dS. This approach requires a special hardware layout for sensors, and the implementation cost may be high for commercial usage.

## 3. The SensorTalk3 Approach

In this section, we first describe the SensorTalk3 architecture and then show how the datasets are collected in this study.

### 3.1. The SensorTalk3 Architecture

**Figure 2:**
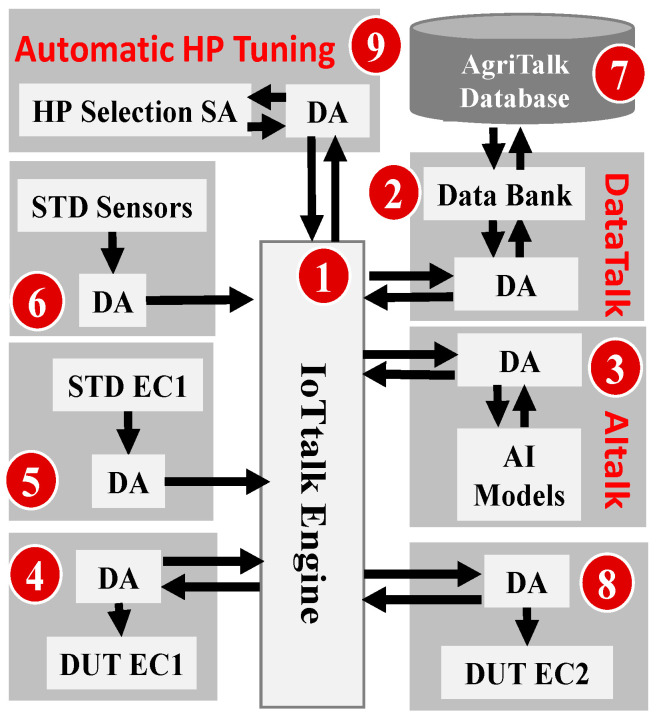
The SensorTalk3 Architecture.

Figure 2 illustrates the SensorTalk3 architecture. The SensorTalk server consists of three components. The IoTtalk engine (Figure 2 (1)) [26] is responsible for interaction with the IoT devices. The DataTalk module (Figure 2 (2)) is responsible for data preprocessing (feature extraction). The AItalk module (Figure 2 (3)) is responsible for AI prediction. An IoT device interacts with the IoTtalk Engine through the Device Application (DA). It is interesting to note that from the viewpoint of the IoTtalk Engine, both DataTalk and AItalk are managed as IoT devices.

The SensorTalk3 server can be deployed in the cloud or installed in an edge node. In the current implementation, the cloud-based SensorTalk3 is deployed in a virtual machine in a commercial cloud at Chunghwa Telecom, the largest Telecom company in Taiwan. The edge-based SensorTalk3 is deployed in a Raspberry Pi4, described in Appendix A.

The EC device under test (DUT EC1 in Figure 2 (4)) is calibrated using the standard EC sensor (STD EC1 in Figure 2 (5)), the standard temperature and moisture sensors (STD Sensors in Figure 2 (6)). In the training phase, the data from devices (4), (5) and (6) are sent to DataTalk through the IoTtalk Engine. After data preprocessing, the extracted features are sent to AItalk through the IoTtalk Engine. Note that the data received by DataTalk may also be sent to the AgriTalk Database (Figure 2 (7)) for archival purposes. Details of data preprocessing and AI modeling are given in the next section.

### 3.2. The Datasets and Data Preprocessing

We have collected the greenhouse data. For every 20 s, we obtain a sample from each of the four sensors to generate a data item. The *i*th data item is a quadruplet $\langle e_{i},E_{i},T_{i},M_{i}\rangle$, where $\left\{e_{i}\right\}$ are obtained from the DUT EC (Figure 2 (4)), $\left\{E_{i}\right\}$ are obtained from the STD EC sensor, $\left\{T_{i}\right\}$ are obtained from the STD temperature sensor, and $\left\{M_{i}\right\}$ are obtained from the STD moisture sensor (Figure 2 (5) and (6)). The set $\left\{E_{i}\right\}$ serves as the labels in the AI models. DataTalk (Figure 2 (2)) computed the mean absolute percentage error (MAPE) between the DUT EC and the STD EC and organized these collected data into 4 datasets:

Dataset 1 collected 20,000 data items during 1 October 2022–17 January 2022, where the EC ranges from 138 to 561 (μS/cm), the temperature ranges from 23.4 to 25.6 (°C), and the moisture ranges from 18.0 to 32.5 (%). The MAPE between the DUT EC and the STD EC is 7.624%.

Dataset 2 collected 11,372 data items during 22 January 2022–26 January 2022, where the EC ranges from 133 to 557 (μS/cm), the temperature ranges from 23.3 to 25.2 (°C), and the moisture ranges from 16.8 to 28.4 (%). The MAPE between the DUT EC and the STD EC is 5.716%.

Dataset 3 collected 44,076 data items during 27 January 2022–2 February 2022, where the EC ranges from 138 to 561 (μS/cm), the temperature ranges from 22.6 to 26.2 (°C), and the moisture ranges from 18.8 to 32.3 (%). The MAPE between the DUT EC and the STD EC is 11.158%.

Dataset 4 collected 36,096 data items during 24 February 2022–3 February 2022, where the EC ranges from 143 to 552 (μS/cm), the temperature ranges from 20.7 to 26.0 (°C), and the moisture ranges from 18.5 to 32.3 (%). The MAPE between the DUT EC and the STD EC is 7.794%.

In these datasets, the MAPEs of the EC without calibration (which is referred to as the “Original” method) range from 5.716% to 11.158%, and therefore, calibration is essential to enhance the accuracy of the EC readings.

We suspect that an EC value is not only affected by the temperature and the moisture but also their change rates. Therefore, DataTalk conducts data preprocessing to create three new features for our AI models. Let $X_{i}$ represents the ith sample of sensor X, where X = e,E,T, or M. Suppose that there are *N* data items in a dataset, where N≥i>w≥0. Let $\delta_{X,i,w}$ denote the change rate of the ith sample of sensor X in the window w. Then, we have

(4) $$\delta_{X,i,w}=\frac{\left|X_{i}-X_{i-w}\right|}{X_{i}}\;\mathrm{and}\;\delta_{X,w}=\frac{\sum_{i=1}^{N}\delta_{X,i,w}}{N}$$

**Figure 3:**
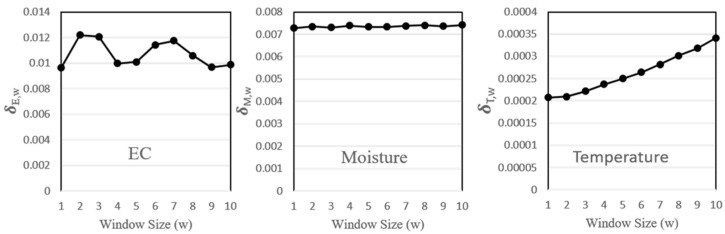
$\delta_{X,w}$ against w, where *X* = (*E*, *M*, *T*).

Figure 3 shows that $\delta_{X,i,w}$ is amplified by w for both moisture and temperature. For EC, $\delta_{X,i,w}$ oscillates with w, which means that we need to choose a small w to reflect recent change trend. In Section 4, we show that w=2 yields the best performance. Therefore, $\delta_{X,i,2}$ are included as the input features in our AI models.

## 4. The AI Models

As we described in Section 2, the EC prediction is affected by temperature, moisture and their change rates. This problem is more appropriately solved via classification-based machine learning models. We consider the following models: The SensorTalk2 model is based on the lookup table [6], XGBOOST [27,28] and Random Forest [29,30]. After collecting and analyzing the data, specifically examining the relationship between each data point of the DUT value and STD value, it becomes apparent that a certain degree of linear correlation exists. Initially, we attempted the conventional approach of using linear regression to calibrate the DUT with Dataset 3, resulting in an unsatisfactory MAPE value of 4.8723%. Upon further inspection of the dataset, it was observed that most data points exhibit a linear correlation, while a few do not show a significant linear relationship. Given the versatility of Random Forest for processing various types of features with linear relationships within the same dataset [29] and the capability of XGBoost in handling numerical features and non-linear relationships between features [28], we decided to employ both algorithms. The experimental results indicate that, in most cases, the MAPEs for XGBoost are generally smaller than those for Random Forest. However, it was noted that Random Forest outperforms XGBoost in specific instances, especially when the dataset comprises mostly linear data along with a few non-linear data points. Considering that Random Forest is adept at processing various types of features with linear relationships within the same dataset, and XGBoost is well-suited for handling numerical features and non-linear relationships between features, we have opted for these two algorithms. Based on these models, we create an ensemble model that utilizes the linear regression method to integrate XGBOOST and Random Forest, which take advantage of the individual machine-learning models to improve the prediction accuracy. The advantage of the classification model is its low time and space complexities in execution. Following the time series of the datasets, we use Dataset 1 for training, Dataset 2 for validation, and Datasets 3 and 4 for inferencing. The input features in our AI models are {*e_i_*, *T_i_*, *M_i_,* $\delta_{e,i,2}$, $\delta_{T,i,2}$, $\delta_{M,i,2}$}, and the labels are $\left\{E_{i}\right\}.$ The output measure of SensorTalk3 is MAPE expressed as

(5) $$\mathrm{MAPE}=\left(\frac{1}{N}\right)\overset{N}{\underset{i=1}{\sum }}\frac{\left|e_{i}-E_{i}\right|}{E_{i}}\;$$

We first select the loss function. The candidates include MSE (Mean Square Error) and RMSLE (Root Mean Squared Logarithmic error) expressed as

(6) $$\mathrm{MSE}=\left(\frac{1}{N}\right)\overset{N}{\underset{i=1}{\sum }}{\left(e_{i}-E_{i}\right)}^{2}$$

and

(7) $$\mathrm{RMSLE}=\left(\frac{1}{N}\right)\overset{N}{\underset{i=1}{\sum }}{\left(\log\left(e_{i}+1\right)-\log\left(E_{i}+1\right)\right)}^{2}$$

Table 1 illustrates the MAPEs of XGBOOST and Random Forest using MSE and RMSLE as loss functions using the default hyperparameters. Since the outliers seldom occur in our sensors, the MSE loss function outperforms the RMSLE function, as indicated in Table 1. We chose MSE as the loss function in our AI Model.

Then, we tune the hyperparameters for AItalk. The set of XGBOOST hyperparameters is expressed as Σ*_XGB_* = {w, n_estimators, max_depth, learning_rate, gamma}. The impact of hyperparameter values on the performance of XGBoost is explained as follows. Increasing “n_estimators” can enhance the model’s complexity and fitting capability, but it also results in higher computational costs and risk of overfitting. “max_depth” defines the maximum depth of each decision tree in a gradient boosting tree. Deeper trees can better capture complex relationships within the training data but are prone to overfitting, especially with limited data. Shallower trees restrict the model’s complexity, helping to prevent overfitting, but may miss some important patterns within the data. The “learning_rate” is used to control the adjustment step size of model weights in each iteration. A lower learning rate makes the model learn more finely, ensuring it does not miss the optimal solution but may require more iterations to achieve peak performance. On the other hand, a larger learning rate speeds up model learning but increases the risk of missing the best solution. The “gamma” value impacts the model’s complexity and helps prevent overfitting. A larger “gamma” value leads to more conservative splitting decisions, simplifying the tree and aiding in preventing overfitting. Smaller “gamma” values allow for more splits, increasing the tree’s complexity, which helps the model fit the training data better but also raises the risk of overfitting [28].

The Random Forest hyperparameter set is expressed as Σ*_RF_* = {w, n_estimators, max_depth, min_samples_split, max_sample}. The impacts of the “n_estimators” and “max_depth” values are similar to those in XGBoost. Additionally, a larger “min_samples_split” value results in a simplified tree, making it more effective in preventing overfitting but potentially at the cost of model flexibility. Conversely, smaller “min_samples_split” values enable more splits, offering better fitting capacity but increasing the risk of overly complex trees, which are more susceptible to overfitting. The “max_samples” hyperparameter in Random Forest controls the fraction of the original dataset assigned to each tree with an optimal performance fraction. This hyperparameter is instrumental in managing model variance and mitigating overfitting issues [29].

Let the default value for a hyperparameter σ be $\sigma_{d}$ and the optimal value be $\sigma_{o}$, where $\sigma_{min}\leq \sigma ,\sigma_{d},{\;\sigma }_{o}\leq \sigma_{max}.\;$The hyperparameter selection (HP Selection) is designed in the following iterative procedure and is implemented as a SA of The automatic HP Tuning device in Figure 2 (9). Let the set of the default hyperparameter values in the AI model X be $\Sigma_{X,d}=\{\sigma_{d}|\forall \sigma \in \Sigma_{X}\}$, where X = XGBOOST or Random Forest. The SA pseudo code is listed below:

**The HP Selection SA**

Line 1. I=0;

Line 2. for (every hyperparameter $\sigma \in \Sigma_{X}$) do {

Line 3. $\Sigma_{0}\leftarrow \Sigma_{X,d};{\;\Sigma }_{1}\leftarrow \emptyset ;$ $\sigma_{o,0}\leftarrow \sigma_{min}$;

Line 4. for ($\sigma_{min}\leq \sigma_{0}\leq \sigma_{max}$) do {

Line 5. Execute the AI model X with the hyperparameter value set $\left\{\sigma_{0}\}\cup^{}\Sigma_{0}-\{\sigma_{d}\right\}$;

Line 6. If (the prediction result with $\sigma_{0}$ is better than the prediction result with $\sigma_{o,0}$) then

Line 7. $\sigma_{o,0}\leftarrow \sigma_{0}$;

        }

Line 8. $\Sigma_{1}\leftarrow \Sigma_{1}\cup^{}\left\{\sigma_{o,0}\right\};\;I\leftarrow I+1;$}

Line 9. while ($\Sigma_{I}\neq \Sigma_{I-1})$ do {

Line 10. for (every hyperparameter $\sigma \in \Sigma_{X}$) do {

Line 11. $\Sigma_{I+1}\leftarrow \emptyset ;$ $\sigma_{o,I}\leftarrow \sigma_{min}$;

Line 12. for ($\sigma_{min}\leq \sigma_{I}\leq \sigma_{max}$) do {

Line 13. Execute AI model X with the hyperparameter value set $\left\{\sigma_{I}\}\cup^{}\Sigma_{I}-\{\sigma_{o,I-1}\right\}$;

Line 14. If (the prediction result with $\sigma_{I}$ is better than the prediction result with $\sigma_{o,I}$) then

Line 15. $\sigma_{o,I}\leftarrow \sigma_{I}$;

}

}

Line 16. $\Sigma_{I+1}\leftarrow \Sigma_{I+1}\cup^{}\left\{\sigma_{o,I}\right\};\;I\leftarrow I+1;$ }

**Figure 4:**
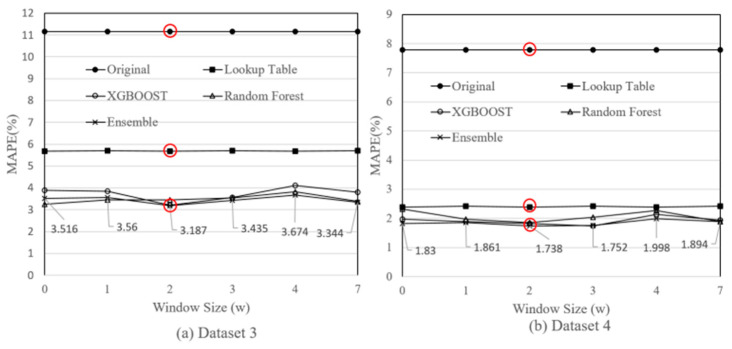
Selection of the change rate window size. (Red circles indicate the optimal value).

In Part 1 (Lines 1–8), for every hyperparameter σ, we execute the AI model X by varying the σ  values in the range [$\sigma_{min},\sigma_{max}$]. For $\sigma^{*}\in \;\Sigma -\left\{\sigma \right\}$, $\sigma_{d}^{*}$ is used in model X (Line 5). The $\sigma_{d}\;$values for XGBOOST are 2 for w, 100 for n_estimators, 6 for max_depth, 0.3 for learning_rate, and 0 for gamma. The $\sigma_{d}\;$values for Random Forest are 2 for w, 100 for n_estimators, None for max_depth, 2 for min_samples_split, and 1.0 for max_sample. If max_depth=None, then the nodes are expanded until all leaves are pure or until all leaves contain less than min_samples_split samples. Figure 4 shows the MAPE against w. This Figure indicates that the default value w=2 turns out to be the optimal value.

**Figure 5:**
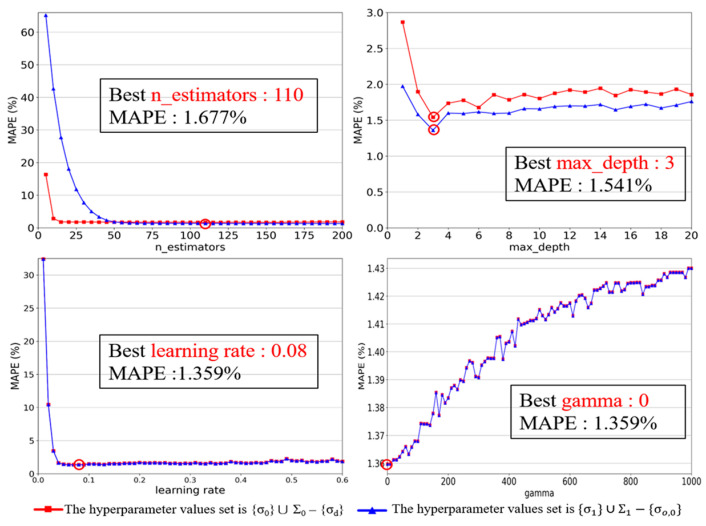
Hyperparameter tuning for XGBOOST. (Red circles indicate the optimal value).

The red square curves in Figure 5 show the MAPE against σ for XGBOOST in Part 1. The $\sigma_{o}$ values selected are 110 for n_estimators, 3 for max_depth, 0.08 for learning_rate, and 0 for gamma. Then, we perform Part 2 (Lines 9–16). Part 2 is the same as Part 1 except that $\sigma_{o}^{*}$ is used instead of $\sigma_{d}^{*}.\;$The blue triangle curves in Figure 5 show the MAPE against σ for XGBOOST in Part 2. The $\sigma_{o}$ values selected are the same as those in Part 1, that is, $\Sigma_{I}=\Sigma_{I-1}$ in Line 9.

**Figure 6:**
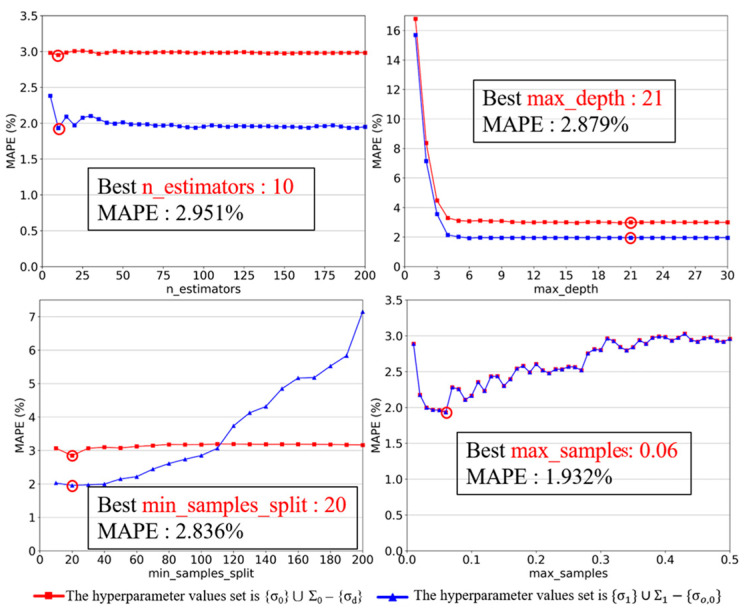
Hyperparameter tuning (tuning) for Random Forest. (Red circles indicate the optimal value).

Therefore, the selected values converge. If $\Sigma_{I}\neq \Sigma_{I-1}$, we repeat Part 2. Similarly, we select the hyperparameters for Random Forest following the same process, and Figure 6 indicates that $\sigma_{o}$ values selected are 10 for n_estimators, 21 for max_depth, 20 for min_samples_split, and 0.06 for max_samples.

SensorTalk3 also performs an ensemble of XGBOOST and Random Forest. The MAPEs of XGBOOST are smaller than Random Forest for most cases. However, we found that Random Forest outperforms XGBOOST on some data. Therefore, SensorTalk3 adopts the ensemble method [31] to yield the best results. Specifically, XGBOOST and Random Forests are used as the base learners. Then, we use linear regression as the meta-learner to combine the predictions of the two base learners, and the ensemble model is trained to improve the performance of the base learners.

## 5. Performance Evaluation

In this section, we first describe the SensorTalk3 performance in terms of the accuracy of calibration. Then, we describe how SensorTalk3 is implemented in Raspberry Pi4 with good time and space complexities.

### 5.1. Accuracy of Calibration

Based on the AI models described in Section 4, the MAPE performance of the original method, lookup table method [6,7], XGBOOST, Random Forest, and SensorTalk3 (Ensemble) are shown in Table 2. The table indicates that SensorTalk3 significantly reduces the MAPEs from 11.159% to 3.187% for Dataset 3, and from 7.792% to 1.738% for Dataset 4.

In Figure 4, if the change rate features are not used (as most previous approaches did), then the MAPEs for Datasets 3 and 4 are 3.516% and 1.83%, respectively. When the change rate with the window size 2 is used, the MAPEs are reduced to 3.18% (for Dataset 3) and 1.738% (for Dataset 4), respectively.

**Figure 7:**
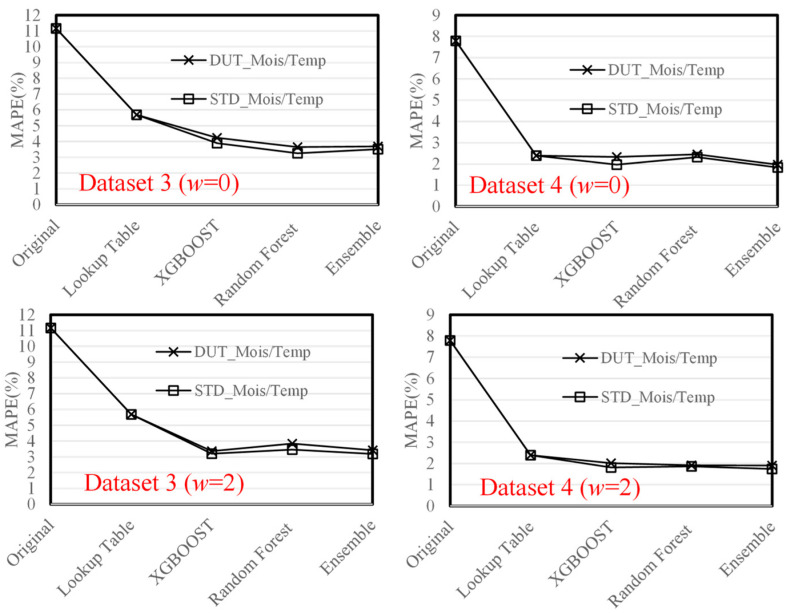
Effect of the accuracies of temperature and moisture.

An interesting question is how the accuracies of temperature and moisture affected the EC prediction. Figure 7 shows the MAPEs for the EC prediction using the STD moisture/temperature sensors and the DUT moisture/temperature sensors, where their error rates for Dataset 3 are 3.51% and 3.69% with w = 0, 3.18% and 3.42% with w = 2, respectively. For Dataset 4, the error rates of the EC prediction using the STD/DUT moisture/temperature sensors are 1.83% and 1.96% with w = 0, 1.73% and 1.90% with w = 2, respectively. Compared to STD moisture/temperature sensors, the DUT moisture/temperature sensors have an average error of 8.3%. The figure indicates that the effect of the accuracies of moisture/temperature is not significant in terms of EC calibration. That is, even if STD moisture/temperature sensors are not used, SensorTalk3 can still calibrate EC well if the errors of the usual temperature/humidity sensors are reasonably small.

### 5.2. Time and Space Complexities of the Edge-Based IoTtalk Engine

In SensorTalk3, the IoTtalk engine is installed in an industry version of Raspberry Pi4 (Broadcom BCM2711, quad-core Cortex-A72 (ARM v8) 64-bit SoC @ 1.5 GHz and Memory: 4 GB LPDDR4), which provides the edge computing solution.

Most edge AI solutions (see [32] and the references therein) perform model training in the network and may further train the edge nodes by using federated learning or transfer learning. Unlike these restricted approaches, the edge-based SensorTalk3 performs real-time training at the edge.

In Figure 2, the data sent from (4)–(6) to the IoTtalk Engine periodically for every 20 s. From the measurement of edge-based SensorTalk3, the time complexity $C_{T,n}$ for processing n consecutive data items in the training phase is expressed as

(8) $$C_{T,n}\approx \;0.06n+249.2\;\left(\mathrm{ms}\right)\;$$

From Equation (8), the execution time for one input data is $C_{T,1}\approx$ 249.26 (ms). In Section 4, SensorTalk3 is an ensemble method of XGBoost and Random Forest. Each of the AI models has 5 hyperparameters to tune. Therefore, when edge-based SensorTalk3 receives one data item, 10 models and the ensemble method are executed to process the data item. The processing time is 249.26 × 11 = 2741.86 ms. The sampling rate of SensorTalk3 is 1/20 s. That is, we have 20 s to process a data item. From the above calculation results, the sampling rate can accommodate the execution of model training. Each model consumes 158 MB of storage, and 4 GB memory suffices to accommodate 158 × 11 = 1.74 GB storage consumption. Therefore, SensorTalk3 is able to collect data and train AI models in parallel at the edge node.

From 10,000 measurements, the time complexity to process one data item in the inference phase is 0.01407 s (with a variance of 0.4007/${\left(0.01407\right)}^{2}$).

The analysis of the time and the space complexities in this subsection shows that we have not used up the time/space resources of the edge node, which proves the feasibility of transfer learning conducted in the edge-based SensorTalk3 solution. Such feasibility is successfully achieved after we have resolved a major challenge of SensorTalk3: the IoTtalk engine is implemented in Python3. When the engine is installed in Raspberry Pi4, the IoTtalk code can be easily broken into and illegally modified to cause malfunctions. To resolve this security issue, we collaborated with Winbond by using the W77Q TrustME^®^ Secure Serial Flash memory. The details are given in Appendix A. We note that transfer learning in the cloud will take extra time before a sensor is calibrated, and during the calibration period, the sensor will produce inaccurate values. With SensortTalk3’s transfer learning in the edge node, we can alleviate this problem.

### 5.3. Calibration Frequency

An important issue for automatic calibration is how to detect when an EC sensor drifts and to perform the AI training procedure in Section 4. One possibility is to periodically perform AI training. A major problem of this approach is that it is difficult to find the optimal frequency to re-train. If we re-train too frequently, then the AI training cost is too high. If we re-train too infrequently, then the EC sensor will report wrong values for a long time before it is calibrated again.

**Figure 8:**
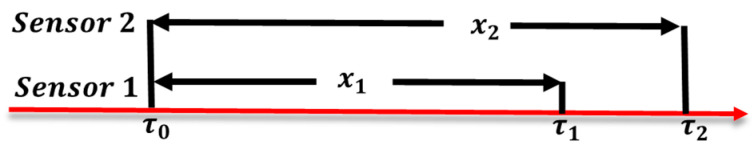
The timing diagram.

Thus, we propose the dual-sensor detection approach that uses two (non-standard) EC sensors for failure detection. Consider the timing diagram in Figure 8.

Initially, both EC Sensors 1 and 2 are calibrated at time $\tau_{0}$ by a standard EC sensor using our AI training procedure. After $\tau_{0}$, both sensors start producing the measured samples. When the SensorTalk server receives the redundant data from these two sensors, we check if they are consistent. If not, then at least one of them needs to be calibrated. Suppose that Sensor 1 drifts at $\tau_{1}\;$and Sensor 2 drifts at $\tau_{2}$, respectively. Let $x_{1}=\tau_{1}-\tau_{0}\;$and $x_{2}=\tau_{2}-\tau_{0}$. It is clearly that we receive consistent results in the time period $x_{min}=\min(x_{1},x_{2})$. After $\tau_{0}+x_{min}$, we will detect inconsistent measures. In Figure 8, $x_{min}=x_{1}$, which means that Sensor 2 is still producing correct data after time$\;\tau_{1.}$ However, we need to calibrate both sensors again at $\tau_{1.}$ Therefore, the frequency for calibration of a sensor is $1/E\left[x_{min}\right]$. In the optimal case (which, unfortunately, cannot be achieved), we only need to calibrate a sensor when it actually drifts, and the frequencies for calibration of Sensors 1 and 2 are $1/E\left[x_{1}\right]$ and $1/E\left[x_{2}\right]$, respectively. Therefore, as compared with the “theorical optimal” overhead, the relative calibration overheads for Sensors 1 and 2 in our approach are

(9) $$O_{1}=\frac{E\left[x_{1}\right]}{E\left[x_{min}\right]}\;\;\mathrm{and}\;O_{2}=\frac{E\left[x_{2}\right]}{E\left[x_{min}\right]}$$

We derive $x_{min}$ and then $O_{1}\;$and $O_{2}$ as follows. From Figure 8, it is clear that

(10) $$E\left[x_{min}\right]=E\left[x_{1}\right]\mathrm{Pr}\left[x_{2}>x_{1}\right]+E\left[x_{2}\right]\mathrm{Pr}\left[x_{1}>x_{2}\right]$$

Suppose that $x_{1}$ and $x_{2}\;$have Erlang distributions. The Erlang distribution is widely used in IoT network modeling [6]. With the shape parameter i and the scale parameter β, the Erlang density function is expressed as

(11) $$f_{E,i}\left(\beta ,x\right)=\frac{\;\beta^{i}x^{i-1}e^{-\beta x}}{\left(i-1\right)!}\;$$

Let the density functions of $x_{1}$ and $x_{2}\;$be $f_{E,i}\left(\beta_{1},x_{1}\right)\;$and $f_{E,j}\left(\beta_{2},x_{2}\right)$, respectively. Then

(12) $$E\left[x_{1}\right]=\frac{i}{\beta_{1}}\;,\;E\left[x_{2}\right]=\frac{j}{\beta_{2}}$$

From Equation (11)

$$E\left[x_{2}\right]\mathrm{Pr}\left[x_{1}>x_{2}\right]\;=\int_{x_{2}=0}^{\infty }\int_{x_{1}=x_{2}}^{\infty }x_{2}f_{E,i}\left(\beta_{1},x_{1}\right)f_{E,j}\left(\beta_{2},x_{2}\right)dx_{1}dx_{2}\;=\int_{x_{2}=0}^{\infty }x_{2}\left[\frac{\beta_{2}^{j}x_{2}^{j-1}e^{-\beta_{2}x_{2}}}{\left(j-1\right)!}\right]\overset{i-1}{\underset{k=0}{\sum }}\left(\frac{\beta_{1}^{k}x_{2}^{k}e^{-\beta_{1}x_{2}}}{k!}\right)dx_{2}\;=\overset{i-1}{\underset{k=0}{\sum }}\left[\frac{\beta_{1}^{k}\beta_{2}^{j}}{k!\left(j-1\right)!}\right]\left[\frac{\left(k+j\right)!}{{\left(\beta_{1}+\beta_{2}\right)}^{k+j+1}}\right]\;\times \int_{x_{2}=0}^{\infty }\left[\frac{{\left(\beta_{1}+\beta_{2}\right)}^{k+j+1}x_{2}^{k+j}e^{-\left(\beta_{1}+\beta_{2}\right)x_{2}}}{\left(k+j\right)!}\right]dx_{2}$$

(13) $$=\overset{i-1}{\underset{k=0}{\sum }}j\left(\begin{array}{c} k+j\\ k \end{array}\right)\left[\frac{\beta_{1}^{k}\beta_{2}^{j}}{{\left(\beta_{1}+\beta_{2}\right)}^{k+j+1}}\right]$$

Substitute Equation (13) into Equation (10) to yield

(14) $$E\left[x_{min}\right]=\overset{i-1}{\underset{k=0}{\sum }}j\left(\begin{array}{c} k+j\\ k \end{array}\right)\left[\frac{\beta_{1}^{k}\beta_{2}^{j}}{{\left(\beta_{1}+\beta_{2}\right)}^{k+j+1}}\right]+\overset{j-1}{\underset{k=0}{\sum }}i\left(\begin{array}{c} k+i\\ k \end{array}\right)\left[\frac{\beta_{2}^{k}\beta_{1}^{i}}{{\left(\beta_{1}+\beta_{2}\right)}^{k+i+1}}\right]$$

Let $\beta_{2}=\alpha \beta_{1}$, then Equation (14) is rewritten as

(15) $$E\left[x_{min}\right]=\overset{i-1}{\underset{k=0}{\sum }}\left(\frac{j}{\beta_{1}}\right)\left(\begin{array}{c} k+j\\ k \end{array}\right)\left[\frac{\alpha^{j}}{{\left(1+\alpha \right)}^{k+j+1}}\right]+\overset{j-1}{\underset{k=0}{\sum }}\left(\frac{i}{\beta_{1}}\right)\left(\begin{array}{c} k+i\\ k \end{array}\right)\left[\frac{\alpha^{k}}{{\left(1+\alpha \right)}^{k+i+1}}\right]$$

From Equations (9), (12) and (15), we have

(16) $$\frac{E\left[x_{min}\right]}{E\left[x_{1}\right]}=\frac{1}{O_{1}}=\overset{i-1}{\underset{k=0}{\sum }}\left(\frac{j}{i}\right)\left(\begin{array}{c} k+j\\ k \end{array}\right)\left[\frac{\alpha^{j}}{{\left(1+\alpha \right)}^{k+j+1}}\right]+\overset{j-1}{\underset{k=0}{\sum }}\left(\begin{array}{c} k+i\\ k \end{array}\right)\left[\frac{\alpha^{k}}{{\left(1+\alpha \right)}^{k+i+1}}\right]$$

From Equations (9) and (12), we have

(17) $$O_{2}=\left(\frac{j}{\alpha i}\right)O_{1}$$

**Figure 9:**
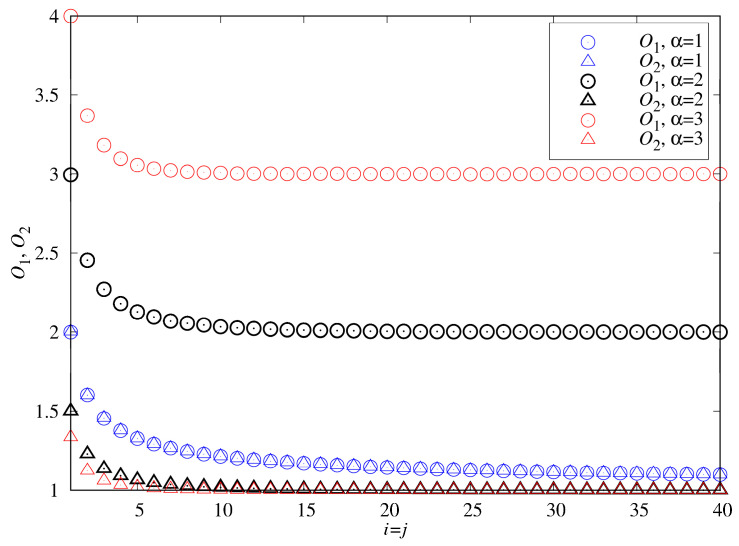
*O*_1_ and *O*_2_ against α, *i* and *j*.

Based on Equations (16) and (17), Figure 9 plots $O_{1}$ and $O_{2}\;$against α, i and j. The figure indicates that the higher the α value, the larger the overhead *O*_1_. A larger value of α implies ×2 is smaller than ×1, which requires Sensor 1 to be frequently calibrated before it actually drifts. Suppose that both Sensors 1 and 2 are produced from the same batch of the manufacture, then we assume that α=1 and i=j. If j=i then Equation (18) is simplified as

(18) $$O_{1}={\left\{\overset{i-1}{\underset{k=0}{\sum }}\left(\begin{array}{c} k+i\\ k \end{array}\right)\left[\frac{\alpha^{i}+\alpha^{k}}{{\left(1+\alpha \right)}^{k+i+1}}\right]\right\}}^{-1}$$

If α=1 then $x_{1}$ and $x_{2}$ have identical Erlang distribution, and Equation (18) is simplified as

(19) $$O_{1}=O_{2}={\left[\overset{i-1}{\underset{k=0}{\sum }}\left(\begin{array}{c} k+i\\ k \end{array}\right)\left(\frac{1}{2^{k+i}}\right)\right]}^{-1}$$

The overheads *O*_1_ and *O*_2_ are the same when α = 1. When α = 2 and 3, *O*_1_ is twice and triple of *O*_2_, respectively. If i=1 then $x_{1}$ and $x_{2}$ are exponential distributions with different means, and Equation (14) is rewritten as

(20) $$O_{1}={\left[\frac{\alpha^{0}+\alpha^{1}}{{\left(1+\alpha \right)}^{1+1}}\right]}^{-1}=1+\alpha$$

If α=1, then $x_{1}$ and $x_{2}$ have identical exponential distributions, and Equation (19) or Equation (20) are simplified as

(21) $$O_{1}=O_{2}=2$$

which gives the mean value analysis [6] to provide the upper bound overheads of our approach. In Figure 9, if α=1, i = 2 then Equation (18) is re-written as

(22) $$O_{1}=O_{2}={\left[\overset{1}{\underset{k=0}{\sum }}\left(\begin{array}{c} k+2\\ k \end{array}\right)\left(\frac{1}{2^{k+2}}\right)\right]}^{-1}=\frac{8}{5}\;$$

If α=1, i = 3 then Equation (18) is re-written as

(23) $$O_{1}=O_{2}={\left[\overset{2}{\underset{k=0}{\sum }}\left(\begin{array}{c} k+3\\ k \end{array}\right)\left(\frac{1}{2^{k+3}}\right)\right]}^{-1}=\frac{16}{11}\;$$

Equations (21)–(23) indicate that as the sensor life is more stable (i.e., the variance is smaller or i is larger), the overheads $O_{1},\;O_{2}$ are smaller. Indeed, Figure 9 shows that the overheads *O*_1_ and $O_{2}$ decrease as i=j increases. If non-standard Sensors 1 and 2 are homogeneous and stable (α=1 and i=j≫1), then $O_{2}=O_{1}\approx 1$, and the extra overhead is negligible.

## 6. Conclusions

SensorTalk3 is an innovative approach for EC sensor calibration, which can be conducted in the cloud or at the edge node. Our study indicated that the soil information (both temperature and moisture sensor data) plays an important role in SensorTalk3, which significantly outperforms the existing lookup table calibration approaches. The MAPE of SensorTalk3 can be as low as 1.738%.

SensorTalk3 is sensitive to the change rate of temperature, moisture and EC in 40 s (i.e., w = 2), and the changes larger or smaller than 40 s have a smaller impact on the calibration. Our study also indicated that the accuracies of moisture/temperature are not significant in terms of EC calibration. Specifically, if the error rates of uncalibrated moisture and temperature sensors are under 8.3%, SensorTalk3 can still calibrate EC well.

SensorTalk3 conducts EC sensor calibration by using XGBOOST and Random Forest. The time/space complexities of these two AI models are lower than complicated deep learning AI models. Therefore, we can perform model training in parallel during data collection at the edge node without GPU acceleration. When the training data are completely collected, AI training is also finished at the edge node. Such an AI training approach has not been found in most edge AI approaches. We also proposed the dual-sensor detection solution to determine when to conduct recalibration. The overhead of this solution is less than twice the optimal detection (which cannot be achieved practically). If the two non-standard sensors are homogeneous and stable (α=1 and i=j≫1), then $O_{2}=O_{1}\approx 1$, then the extra overhead is negligible. Traditional approaches necessitate the training of calibration AI models in the cloud. A major contribution of SensorTalk3 is that it revolutionizes this process by introducing a groundbreaking feature: transfer learning can now be conducted directly on the edge node. This cutting-edge capability becomes especially advantageous in the context of agricultural settings, where numerous farming sensors are deployed throughout vast fields. By enabling local transfer learning on inexpensive edge nodes, SensorTalk3 offers farmers a significantly more cost-effective solution for optimizing their systems. Additionally, there is a delay in achieving calibration and obtaining accurate readings when implementing transfer learning in the cloud. This can lead to inaccurate values being generated by the sensor during the calibration period. However, by utilizing transfer learning in the edge node with SensortTalk3, we can mitigate this issue.

SensorTalk3 has been deployed in commercial farm fields (refer to Figure 1b). Similar to other AI applications, when SensorTalk3 is utilized in new farm fields with varying conditions such as temperature range, soil type, relief, etc., standard transfer learning is necessary. This learning process can be conducted seamlessly and automatically through the IoT configuration of SensorTalk3 (refer to Figure 2).

## Figures and Tables

**Table 1:** Loss function selection.

Loss Function	MAPE	
	XGBOOST	Random Forest
MSE	1.714%	2.981%
RMSLE	54.701%	3.180%

**Table 2:** (Ensemble) and other AI models.

Dataset.	MAPE				
	Original	Lookup Table	XGBOOST	Random Forest	Ensemble
Dataset3	11.159%	5.690%	3.203%	3.453%	3.187%
Dataset4	7.792%	2.393%	1.818%	1.861%	1.738%

**Figure A1:**
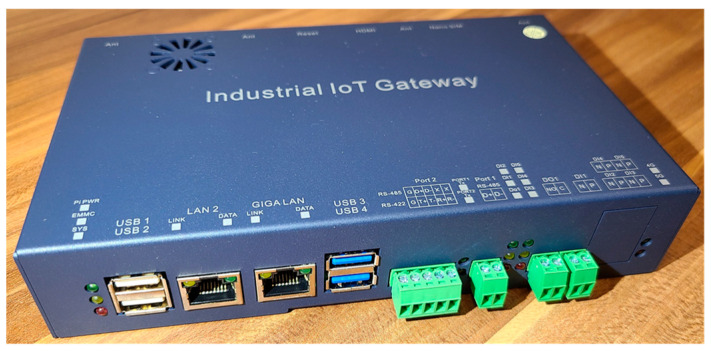
Secured edge-based SensorTalk3 server.

**Figure A2:**
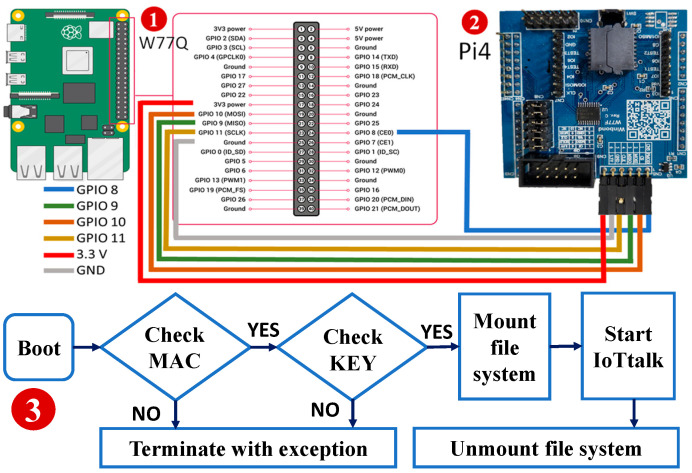
Connecting W77Q and Pi4 for IoTtalk engine.

**Figure A3:**
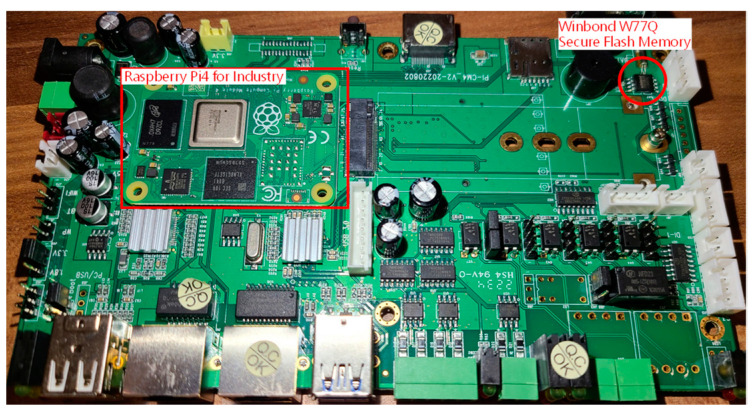
The edge-based IoTtalk circuit board.

## Appendix A

The edge-based SensorTalk3 solution protected by W77Q TrustME^®^ secure serial flash memory. With W77Q supported by Winbond, the IoTtalk Python code is well protected in an industrial Raspberry Pi4 to avoid illegal access. The edge-based SensorTalk3 server is illustrated in Figure A1.

W77Q TrustME^®^ Secure Serial Flash memory (Figure A2 (1)) provides a secure storage solution for Pi4 (Figure A2 (2)) with limited space, pins and power, which meets Common Criteria EAL2 Security Certification requirements [33]. W77Q is a drop-in replacement for standard Serial NOR Flash Memory devices, offering security, flexibility, and performance well beyond ordinary NOR Flash Memory devices. It provides support for execution in place, cryptographic key distribution, and secure data storage. W77Q features sophisticated cryptographic encryption of the communication channel, personalization of each device with unique keys, cryptographic read and write locks, protection of data integrity, secure firmware update, root of trust functions, and secure read, write and erase operations. W77Q supports single, dual, and quad SPI as well as QPI modes of operation, running at up to 133 MHz. Dual Transfer Rate (DTR) is supported at rates up to 66 MHz.

In the proof of concept (POC) stage, we connected W77Q with Pi4 through individual development boards, and executed the startup procedure for the IoTtalk engine as illustrated in Figure A2 (3). The Python code of IoTtalk is protected in W77Q with the 256-bit SHA256 key. When the system is turned on, the startup procedure first check if the MAC address and the cryptographic key of the Pi4 are correct. If so, the protected IoTtalk code is loaded into the Pi4 storage for execution, and then the security flash is disconnected [34]. After the POC stage, W77Q and Pi4 were integrated into a printed circuit board (Figure A3) and become a commercial product, as illustrated in Figure A1.
